# The lymphatic system and sentinel lymph nodes: conduit for cancer metastasis

**DOI:** 10.1007/s10585-021-10123-w

**Published:** 2021-10-15

**Authors:** Stanley P. Leong, Alexander Pissas, Muriel Scarato, Francoise Gallon, Marie Helene Pissas, Miguel Amore, Max Wu, Mark B. Faries, Amanda W. Lund

**Affiliations:** 1 California Pacific Medical Center and Research Institute, San Francisco, CA USA; 2 Department of Visceral Surgery General Hospital of Bagnols sur Cèze and of Anatomy Faculty of Medicine of Montpellier, Bagnols sur Ceze, Montpellier, France; 3 Vascular Anatomy Lab. III Chair of Anatomy, Faculty of Medicine, Buenos Aires University, Buenos Aires, Argentina; 4 Phlebology and Lymphology Unit. Cardiovascular Surgery Division, Central Military Hospital, Buenos Aires, Argentina; 5 California Pacific Medical Center, San Francisco, USA; 6 The Angeles Clinic and Research Institute A Cedars-Sinai Affiliate, Los Angeles, CA USA; 7 Ronald O. Perelman Department of Dermatology, Department of Pathology, and NYU Langone Laura and Isaac Perlmutter Cancer Center, NYU Grossman School of Medicine, New York, NY 10016 USA; 8 University of California, San Francisco, San Francisco, CA USA

**Keywords:** Lymphatic system, Sentinel lymph nodes, Cancer metastasis and the lymphatic system

## Abstract

The lymphatic system is a complicated system consisting of the lymphatic vessels and lymph nodes draining the extracellular fluid containing cellular debris, excess water and toxins to the circulatory system. The lymph nodes serve as a filter, thus, when the lymph fluid returns to the heart, it is completely sterile. In addition, the lymphatic system includes the mucosa-associated lymphoid tissue, such as tonsils, adenoids, Peyers patches in the small bowel and even the appendix. Taking advantage of the drainage system of the lymphatics, cancer cells enter the lymphatic vessels and then the lymph nodes. In general, the lymph nodes may serve as a gateway in the majority of cases in early cancer. Occasionally, the cancer cells may enter the blood vessels. This review article emphasizes the structural integrity of the lymphatic system through which cancer cells may spread. Using melanoma and breast cancer sentinel lymph node model systems, the spread of early cancer through the lymphatic system is progressive in a majority of cases. The lymphatic systems of the internal organs are much more complicated and difficult to study. Knowledge from melanoma and breast cancer spread to the sentinel lymph node may establish the basic principles of cancer metastasis. The goal of this review article is to emphasize the complexity of the lymphatic system. To date, the molecular mechanisms of cancer spread from the cancer microenvironment to the sentinel lymph node and distant sites are still poorly understood and their elucidation should take major priority in cancer metastasis research.

## Introduction

### Stanley P Leong

In the pre-sentinel node era, it was assumed that cancer would spread from the primary site to the regional lymph nodes first, then to the distant sites. Thus, Halstad promoted radical mastectomy with a complete axillary lymph node dissection for breast cancer [1] and Snow championed *en bloc* resection of the primary melanoma along with a radical regional lymph node dissection [2, 3]. The importance of lympatic system to cancer metastasis has been emphasized and described in detail by Haagensen et al. [4]. The impact by Halsted was so profound that radical lymph node dissections became standard for different types of cancer such as esophagus, gastric, colon, gynecological cancers and others. Despite adequate resection of the primary site and radical regional lymph node dissection, systemic recurrence still occurs. Thus, Cady maintained that the lymph node was the marker but not the governor of cancer spread [5]. The dilemma of assessing the regional lymph node and yet to avoid a radical lymph node dissection was resolved by the work of Cabanas [6] using the penile model and Morton [7] using the melanoma model with their pioneering work in the development of the sentinel lymph node (SLN) concept. Subsequent clinical trials in melanoma [8] and breast cancer [9] have established that SLN biopsy is a reliable staging procedure for the regional nodal basin. Thus, a radical lymph node dissection with increased morbidity can be avoided if the SLN biopsy is negative.

In the post-sentinel node era, we have learned that, in most cases such as melanoma and breast cancer, cancer spreads in an orderly process [10] from the primary site to the SLNs primarily and then beyond to the distant sites. The anatomy and physiology of cancer spread is further defined by the patterns of spread of primary cancer to the SLNs.

In this review article, we want to further define the anatomy of the lymphatic system in selected anatomy sites. Alexander Pissas, Muriel Scarato, Francoise Gallon and Marie Helene Pissas describe the lymphatic drainage of the stomach and pancreas based on surgical and cadaveric dissections. Using cadaveric specimens, Miguel Amore defines the lymphatics of the upper and lower extremity to the axilla and groin respectively. Max Wu studies the lymphatic system and SLNs using different imaging modalities. Beyond melanoma, Mark Faries summarizes the application of sentinel node concept and surgery to assess the status of several types of cancer. Amanda Lund tracks the trafficking of lymphocytes through the lymphatic vessels as well as the relationship between tumor-associated vessels and immune surveillance. Summary and perspectives of the lymphatic system and SLNs in relation to cancer metastasis are given by Stanley Leong.

## The lymphatic drainage of the stomach and pancreas: study on corpses and on the living

### Alexandre Pissas, Muriel Scarato, Francoise Gallon and Marie Helene Pissas

#### The lymphatic drainage of the stomach

Rouviere, Cunéo and Delamare have described three areas of drainage of the stomach: (1) the left gastric drainage close to the lesser curvature; (2) the splenic drainage system corresponding to the gastric fundus; and (3) the hepatic lymphatic drainage [11, 12]. This last area is the largest network and includes the most important part of the greater curvature and the antral pyloric portion of the stomach.

**Fig. 1:**
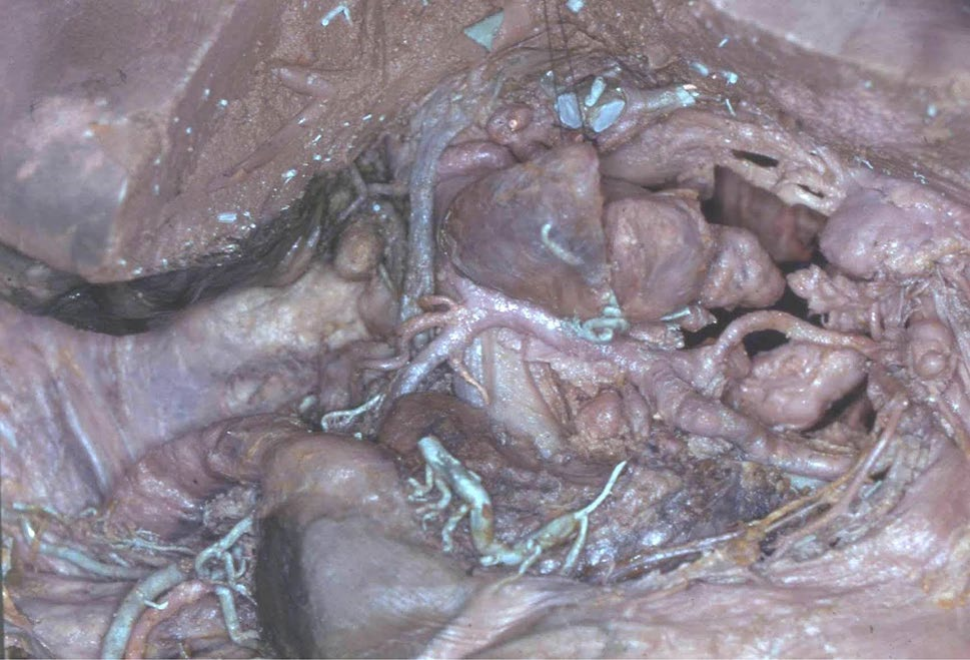
Dissection of lymph nodes of stomach without injection

**Fig. 2:**
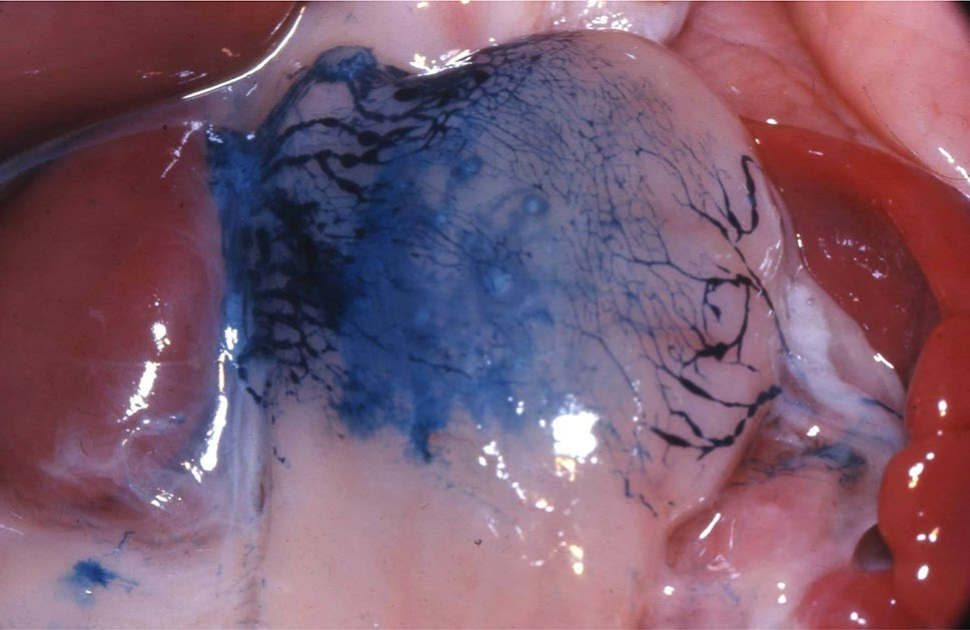
Injection of lymph vessels of the stomach with coloured cedar oil

**Fig. 3:**
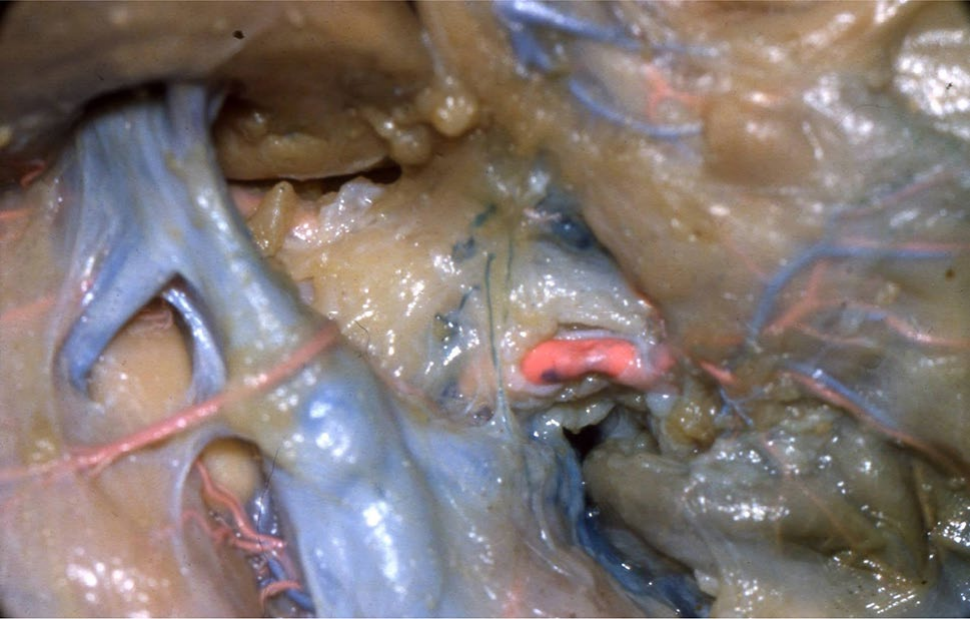
Lymph vessels in the right retroportal process of pancreas

**Fig. 4:**
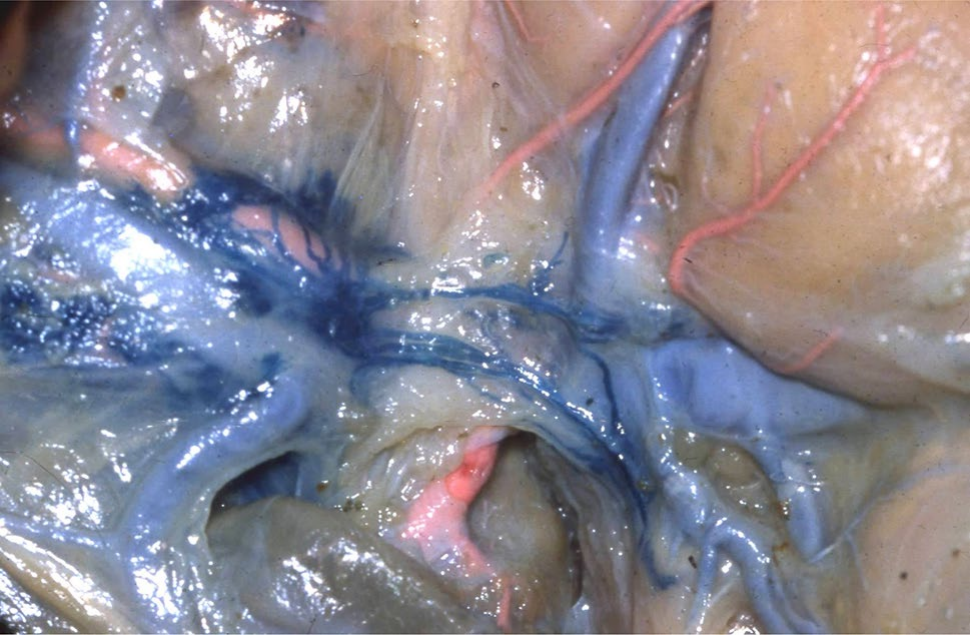
Lymph vessels in the left lateroportal process of pancreas

We have studied the anatomy of the lymphatic drainage of the stomach and pancreas for the past 30 years. In order to appreciate the lymphatic system of these digestive organs, we have used the specimens from cadaveric dissections and tracked the lymphatic drainage of these organs with vital dyes during surgery [13]. We have dissected 20 cadavers without injection and 210 cadavers with injection of Papamiltiades’ solution (colored cedar oil) and 55 cadavers with colored china wood oil (Figs. 1, 2, 3 and 4). Forty patients were injected with Ultra Fluid Lipiodol during fibroscopy and vital staining dye, Pontmine Sky Blue6 BX (Gurr), during surgery. Our findings suggest that the splenic area is less important than the left gastric and hepatic areas, and that the superposition of these three areas is vital. These findings suggest that cancer may spread through the lymphatic system in the middle part of the stomach, in either the left gastric or splenic or hepatic chain. We have established the superposition of these three major areas, in that the middle part of the stomach drains through the left gastric, splenic, or the hepatic channel with the direction of the lymphatic drainage in the direction of the valves on the lymphatic subserous vessels.

A recent review of the lymphatic system of the stomach by Lirosi et al. shows that the lymphatic drainage system of the stomach is complex [14]. The review emphasizes the surgical anatomic orientation and follows the recommendations of the Japanese Research Society for Gastric Cancer [15].

**Fig. 5:**
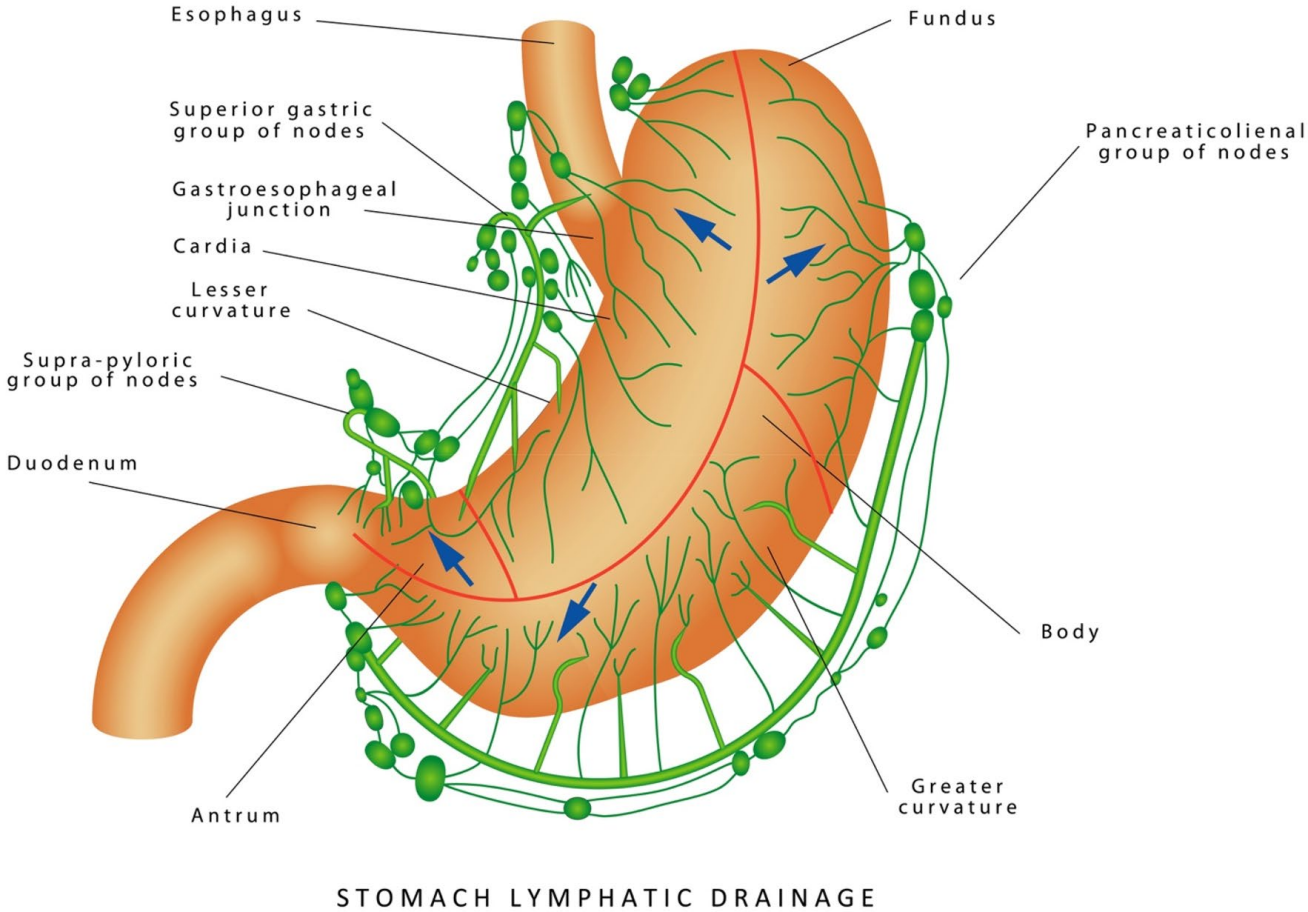
Lymphatic drainage of the stomach from Google Image View. The gastric lymphatic drainage system is complex as discussed in the text

Cadaver models may lack precision because cadaveric studies depend on the direction of the needle and syringe administered by the anatomist upon injection of the colored cedar oil. Therefore, cadaveric findings of the lymphatic system should be supplemented by the flow of the vital dye during surgery. Taken together with the literature and our cadaveric and surgical findings, we can conclude that the lymphatic drainage of the stomach is multiplex and overlapping of the pathways is not infrequent as shown in Fig. 5.

#### The lymphatic drainage of the pancreas

Based on cadaveric injection of pancreas in 12 adults, 15 stillborn babies and 3 infants with Indian Ink, Evans and Ochsner have found splenic, inferior pancreatic, anterior and posterior duodenopancreatic drainage systems [16] without studying the posterior pathways.

**Fig. 6:**
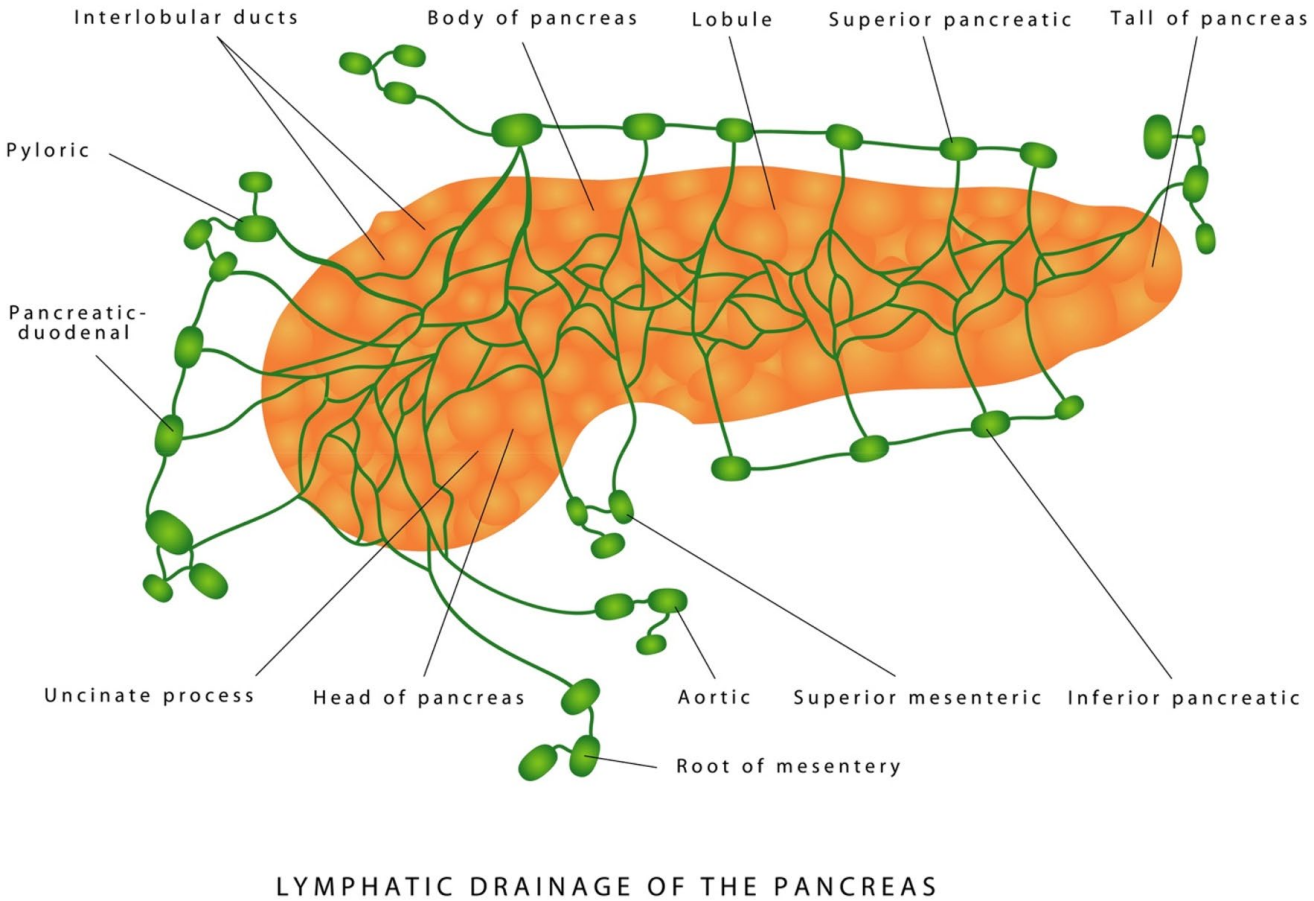
Lymphatic drainage of the pancreas from Google Image View. As described in the text, the pancreatic lymphatic drainage system is complex

A recent review of the lymphatic system of the pancreas by Cesmebasi et al. [17] shows that the pancreatic lymphatic system is complex with an extensive network of lymphatic channels and vessels and nodes draining the head, neck, body and tail of the pancreas (Fig. 6). This complicated lymphatic system of the pancreas has a direct association with the clinical outcomes of pancreatitis and spread of pancreatic cancer [17]. We suggest that the lymphatic system of the pancreas is involved in the pathogenesis of chronic and acute pancreatitis. The lymphatic system, therefore, acts like a safety valve against the progression of acute pancreatitis, for enzymes are macroproteins and are absorbed only by the lymphatic system.

**Fig. 7:**
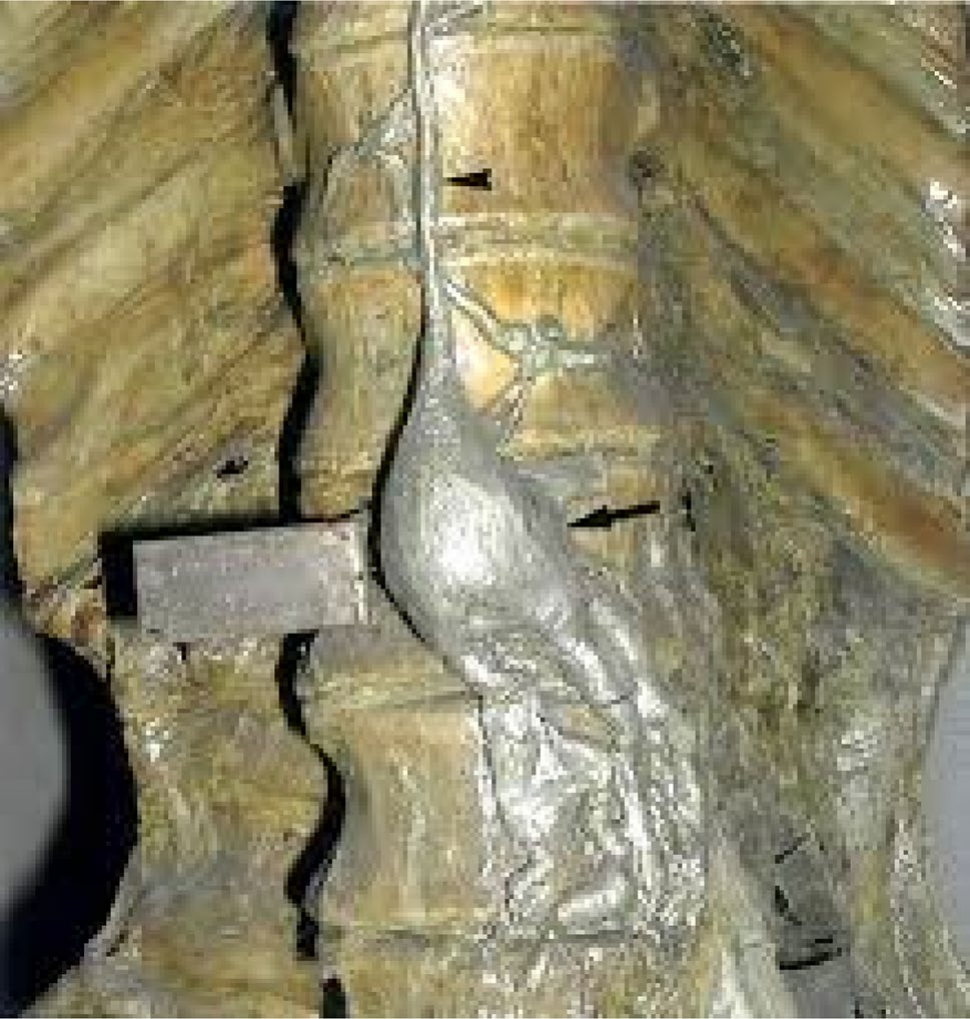
Photograph of a preserved cadaveric specimen shows a large cisterna chyli sac (arrow) anterior to the vertebra L1 and the proximal thoracic duct (arrowhead), afferent trunks consisting of the intestinal, left and right lumbar trunks that join with the inferior part of the cisterna. Also, intrathoracic lymphatic tributaries of the thoracic duct are noted [19]. Permission to use this image and the legend of this figure was obtained from the journal Radiological Society of North America. Published Online: May 01, 2004; 10.1148/rg.243035086)

**Fig. 8:**
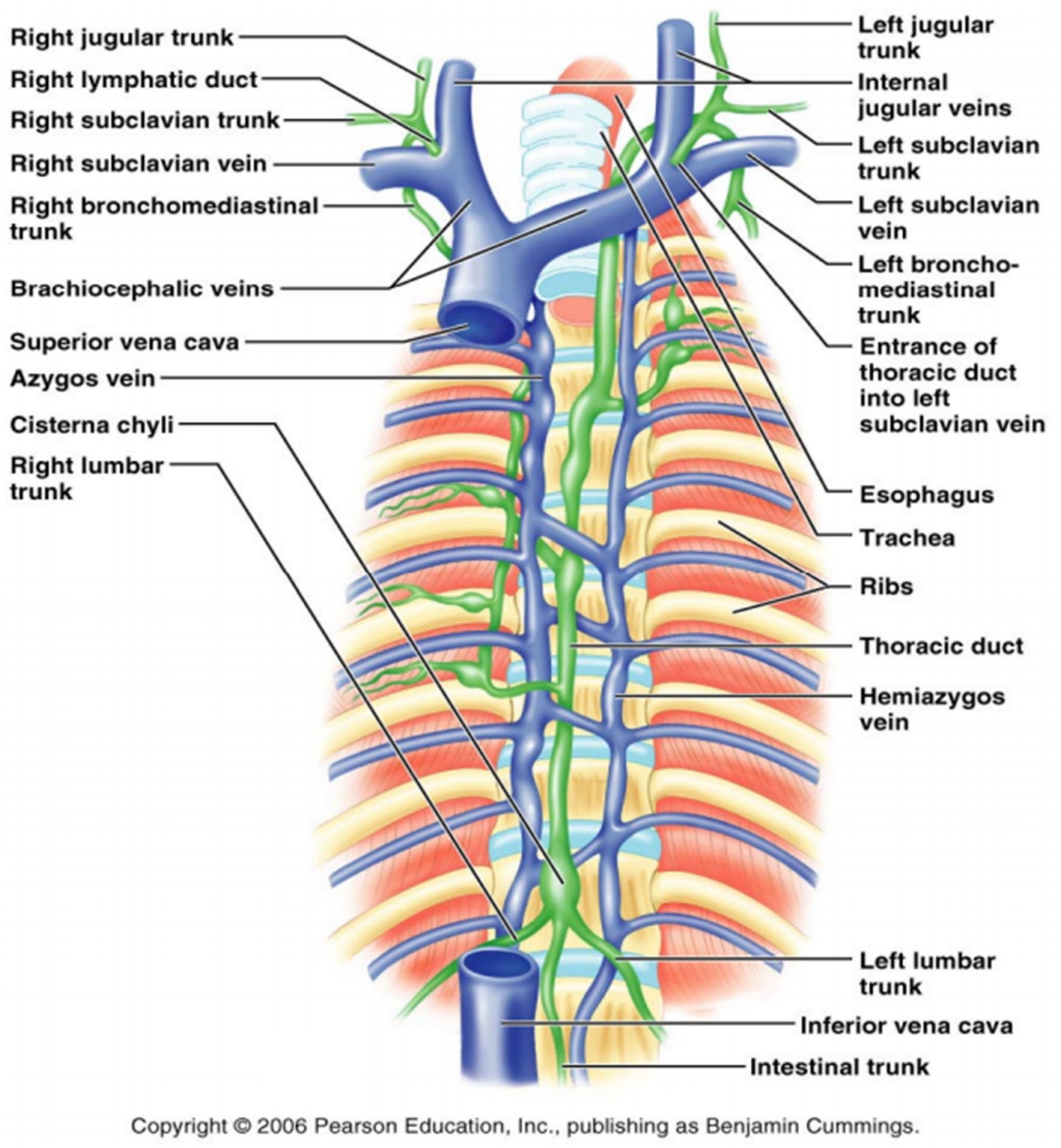
The lymphatic drainage from the spleen, the stomach, pancreas, and liver into the intestinal trunk. The retropertoneal organs such as the kidney, ovary and testicles drain into the right and left lumbar trunks. The pelvic structures including prostate, bladder, uterus and rectum as well as the lower extremities drain into the iliac nodal chains and join with the lumbar trunks. Lymph flows from the intestinal trunk and both lumbar trunks into the cysterna chyli (Fig. 7) and finally to the thoracic duct, which drains into the left internal jugular vein. The lymphatic system from the head and neck (not shown) drains into the left and right jugular lymphatic trunks with the left draining into the thoracic duct and the right into the right subclavian vein. The lymphatic system of the right upper extremity drains into the right subclavian trunk, which returns the lymph at the junction of the right internal jugular and subclavian veins while the left upper extremity drains into the left subclavian trunk, which drains directly into the thoracic duct. The upper right portion of the body drains into the right lymphatic duct, which returns the lymph through the right subclavian vein. The upper left portion of the body drains into the thoracid duct, which returns the lymph into the left subclavian vein. On a daily basis, 17 L of the 20 L of the blood at the arteriovenous capillary junctions return through the venous capillary circulatory system. About 3 L of fluid without cellular components of the blood escape into the extracellar space and drain into the lymphatic vessels as lymph fluid carrying the cellular debris, macromolecules of proteins, excess water and toxins. Lymphadema results when the extracellular fluid is not adequately drained. The lymph fluid is filtered through multiple lymph nodes and finally drain into the thoracic duct or subclavian veins as sterile lymph fluid to rejoin the vascular circulation. Permission to use this image was obtained from Pearson Education, Inc

The concepts of Descomps and Turnesco [18] brought about great progress in the recognition of abdominopelvic drainage of lymph. It is evident that cysterna chyli is not the origin of the thoracic duct but it represents a dilated sac leading into the thoracic duct [19] (Fig. 7). The intestinal trunk and two lumbar trunks flow into the cysterna chyli, which is a retroperitoneal structure and it receives fatty chyle containing lipid products of digestion from the intestines [20]. Thus, the cysterna chyli picks up the drainages from the abdominal and pelvic organs acting as a conduit to drain the abdominopelvic lymph into the thoracic duct (Fig. 8).

## Lymphatics of the axilla and groin based on cadaveric dissections

### Miguel Amore

#### Axillary lymph nodes

The axillary lymph nodes represent the center of the lymphatic drainage of the upper limbs and the mammary gland. We have adopted the classification proposed by Caplan [21], in relation to the venous drainage of the axilla, describing the following four principal chains:

#### Lateral mammary chain

These nodes are found proximal to the axillary vessels, on the anterior walls of the axilla, and they follow the external mammary vessels between the skin of the anterior and posterior regions of the thorax. Its efferent vessels are directed toward the apex of the axilla to drain its lymph into the superior thoracic and axillary chain, which form the traditionally named infraclavicular group.

#### Superior thoracic chain

These nodes reside on the internal wall of the axilla, behind the pectoral muscles, and follow the superior thoracic vessels. The efferent lymphatic vessels of this chain drain their lymph into the axillary vein chain.

#### Subscapular chain

**Fig. 9:**
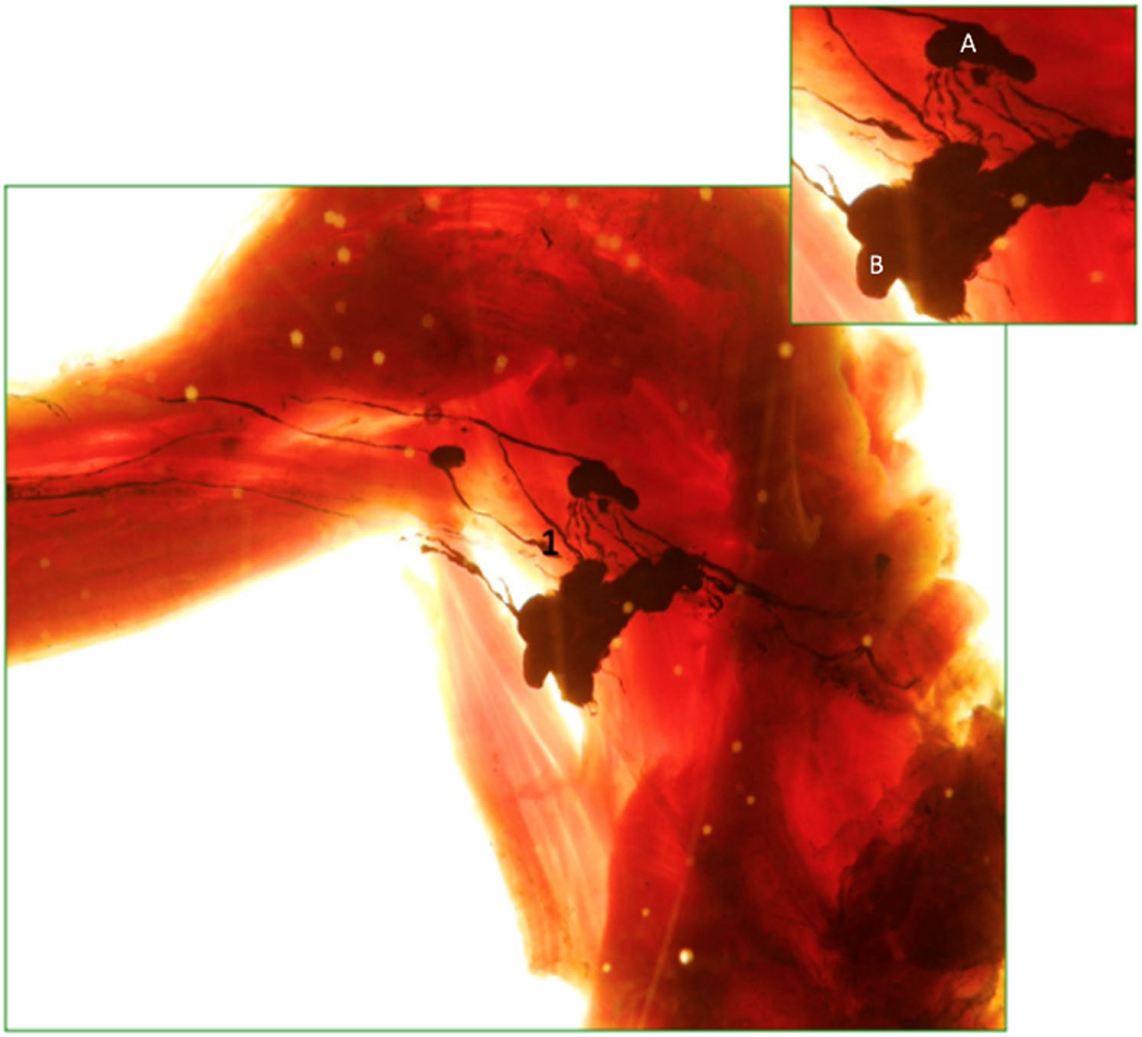
Axillary lymph nodes. **A**. Lateral Mammary chain. **B**. Subscapular chain

These are satellite nodes of the subscapular vessels. They receive drainage of the posterior wall of the thorax and, in a lower percentage, the lymphatic drainage from the anterior and internal thoracic wall (Fig. 9). The efferent of this chain is directed to the external mammary chain or to the apex of the axilla, to drain at the level of the axillary vein.

#### Axillary vein chain

This chain of nodes is located in the upper part of the armpit, at the base to the pectoral axillary apex. This chain includes eight to ten nodes as well as four secondary chains: anterior, posterior, superior, and inferior. This chain receives the lymphatic drainage from all the regions of the upper limbs, from the anterior and posterior wall of the thorax, as well as from the rest of the vertical chains of the axilla. The efferent vessels, generally, follow the axillary vein to reach ultimately to the jugular–subclavian venous angle of the thoracic duct [22–27] (Fig. 9).

#### Derivative lymphatic pathways

It is important to highlight the presence of the derivative lymphatic pathways of the upper limbs, which are those lymphatic vessels that do not reach the axillary lymph nodes. These include the cephalic (Mascagni) [26], the delto-tricipital (Caplan) [22, 26], and the intra-axillary (Ciucci) vessels [22, 26]. Although these alternate lymphatic vessels are not the principal lymphatic drainage toward the axilla, they might have a very important role for the rehabilitation of the patient with lymphedema. It is vital to know their anatomy so that they might be preserved [22, 26].

#### Anatomical and clinical remarks

The predominant lymphatic drainage pathway from the breast is toward the axilla. Axillary node dissection is a standard surgical treatment for select and specific breast cancer patients with positive nodes. Unfortunately, the incidence of arm lymphedema is quite real and is certainly affected by surgical resection as it is reported in about 20–30% of patients undergoing axillary lymph node dissections [28]. Currently, the SLN biopsy has become a widely used method for surgical staging of axillary lymph nodes in breast cancer. A less invasive operation, nevertheless, the incidence of arm lymphedema after SLN biopsy is below 5% [28]. Optimal sites of dye or colloid injection have yet to be defined for SLN biopsies. Intradermic, subareolar, peritumoral, and intra-tumoral dye injection sites have all been reported, though there is no consensus regarding the optimal dye injection site to identify the SLN. The determination of an ideal injection site is derived more from clinical experience than from anatomical studies, as Suami et al. have suggested [29]. Those who injected in the subareolar region were following the concept of the subareolar plexus described by Sappey in 1874 [30]. This plexus was considered the center of all lymphatic drainage in the mammary gland, no matter if the tumor resided in any part of it. But in 1959, Turner-Warwick suggested Sappey might have mistaken a milk duct for a lymphatic vessel because he could demonstrate dye or isotope drainage from the tumor site to the axilla that bypassed the subareolar plexus completely [31]. Recent work studying lymphatic breast drainage in cadavers suggests injecting close to the primary tumor is the most effective way to identify the SLN [29, 31].

#### Inguinal nodes

**Fig. 10:**
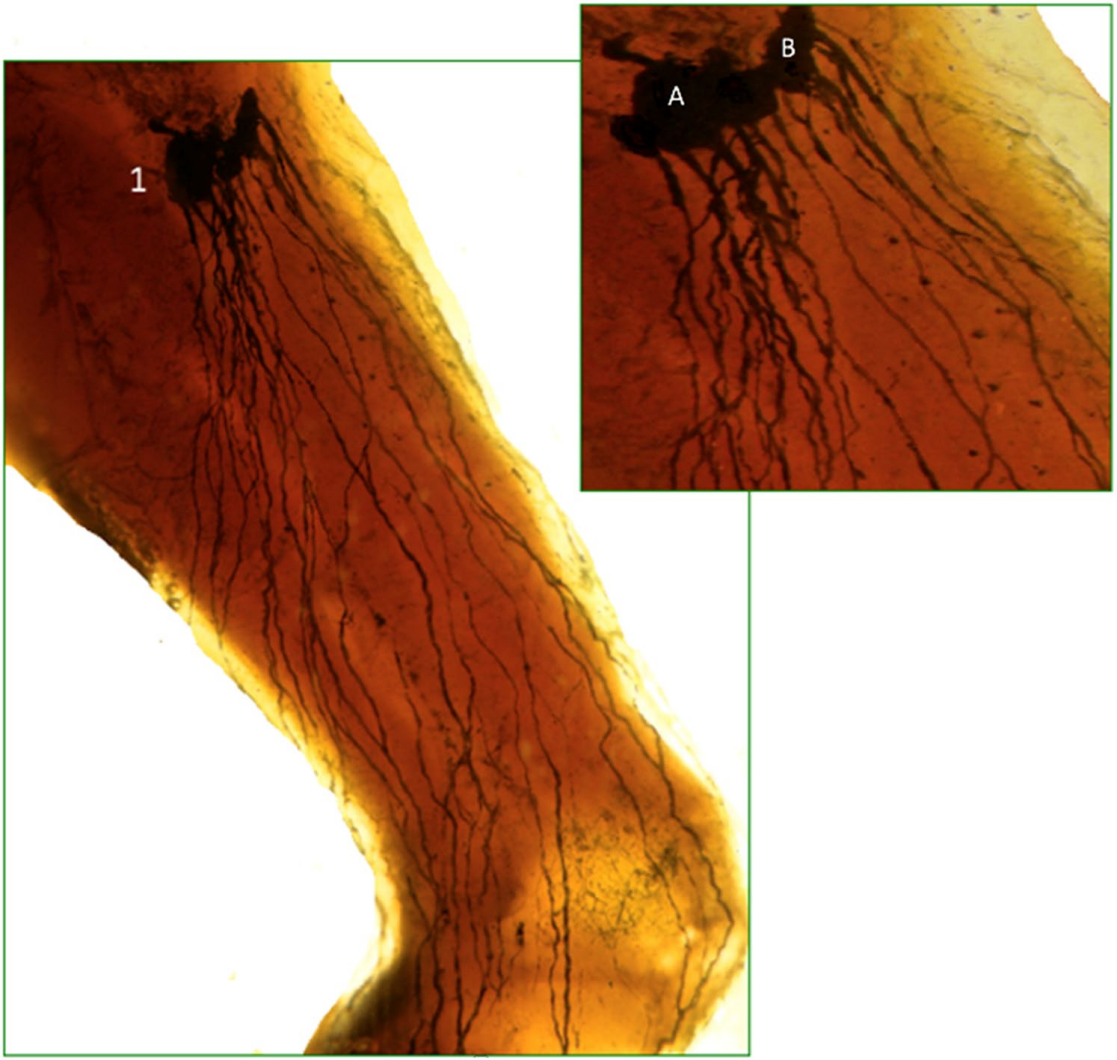
Superficial lymphatic network of the limb. (1) Inguinal nodes. inferior groups. A. Internal saphenous. B. Accessory saphenous. To visualize the lymphatic vessels and lymph nodes in both Figs. 8 and 9, diaphanization technique (Spalteholz) [34] was used to avoid injury to the lymphatic vessels and lymph nodes. After fixation with 4% formaldehyde, the specimen was dehydrated with alcohol and then immersed in xylol, which changes the refractive index and generate a 3D image

These nodes receive lymph from lower limbs, external genitals, the anal margin, and the lower abdominal wall (Fig. 10). There are approximately 10 or more nodes, all located at the superficial fascia. From the thirteenth century to date, a number of authors have described the inguinal nodes and have proposed different nomenclatures to classify the existing node groups, but the one that has stood the test of time is the one proposed by Quènu and Lejars [32]. They classified the groups using two imaginary lines, one perpendicular and one vertical, in relation to the axis of the great saphenous vein. This classification allows these nodes to be grouped in four groups and a central group [22, 25]. In our research, we have divided, according to arch tributaries of the great saphenous vein, into three superior and two inferior groups: external superior group or iliac circumflex, middle or upper abdominal subcutaneous, superior internal or external pudendal, inferior internal or internal saphenous, and inferior external or accessory saphenous. The inguinal lymph nodes drain preferably to the Cloquet lymph node [33], which is located under the inguinal ligament and is the most inferior node of the external iliac chain. The Cloquet lymph node belongs to the medial group of the external iliac chain [30]. External iliac nodes are not included in this classification.

## Imaging of lymph nodes

### Max Wu

Clinical imaging of lymph nodes may be divided into anatomic and functional modalities. Computed tomography (CT), ultrasound (US), and magnetic resonance imaging (MRI) provide detailed anatomic or morphologic evaluation of lymph nodes, while positron emission tomography (PET) and lymphoscintigraphy are functional imaging techniques.

**Fig. 11:**
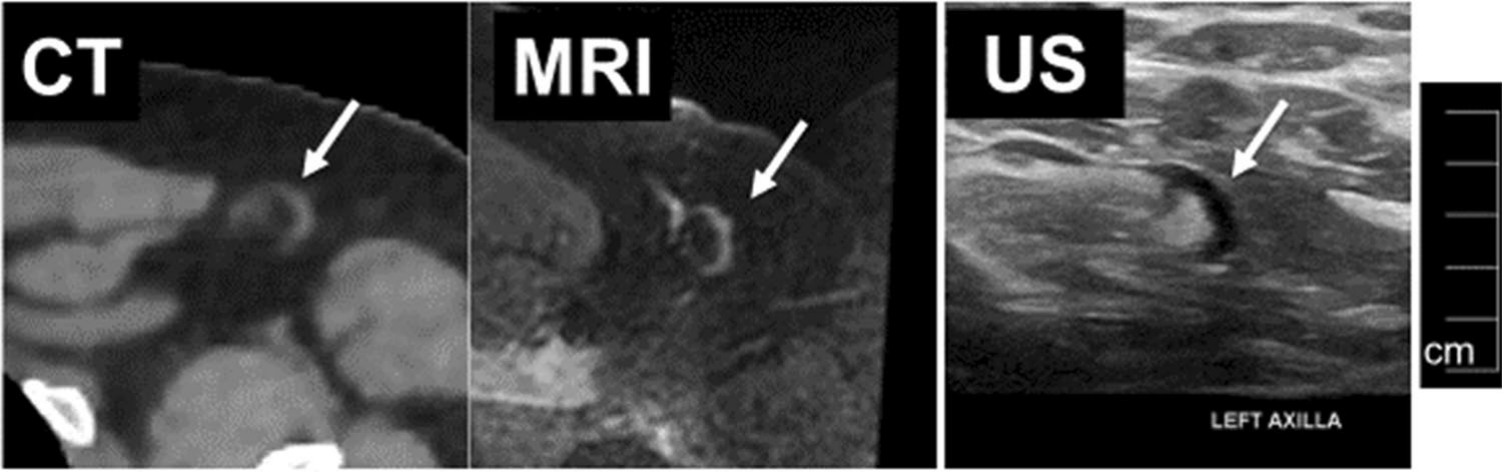
The same normal left axillary node by CT, MRI, and US. All modalities demonstrate the thin cortex (white arrow) and normal fatty hilum

Normal lymph nodes have an elongated or reniform shape characterized by a long axis (along the longest dimension of the node) and short axes (perpendicular to the long axis). Lymph node size is expressed as the maximum short-axis dimension, a less variable measurement than the long-axis dimension. Ten millimeters is often used as the upper limit of normal for the maximum short-axis dimension for lymph nodes in the mediastinum [35] and in other sites (Fig. 11). Of course, pathologic nodes can be smaller than 10 mm, and normal nodes can be larger. In other locations, such as the internal thoracic (internal mammary) basin, nodes are typically smaller and a smaller size cut-off applies. Size criteria for other basins have a more limited evidence basis [36]. The 10 mm short-axis size criterion is widely accepted and has been incorporated into the Response Evaluation Criteria in Solid Tumors (RECIST) version 1.1 [37, 38].

**Fig. 12:**
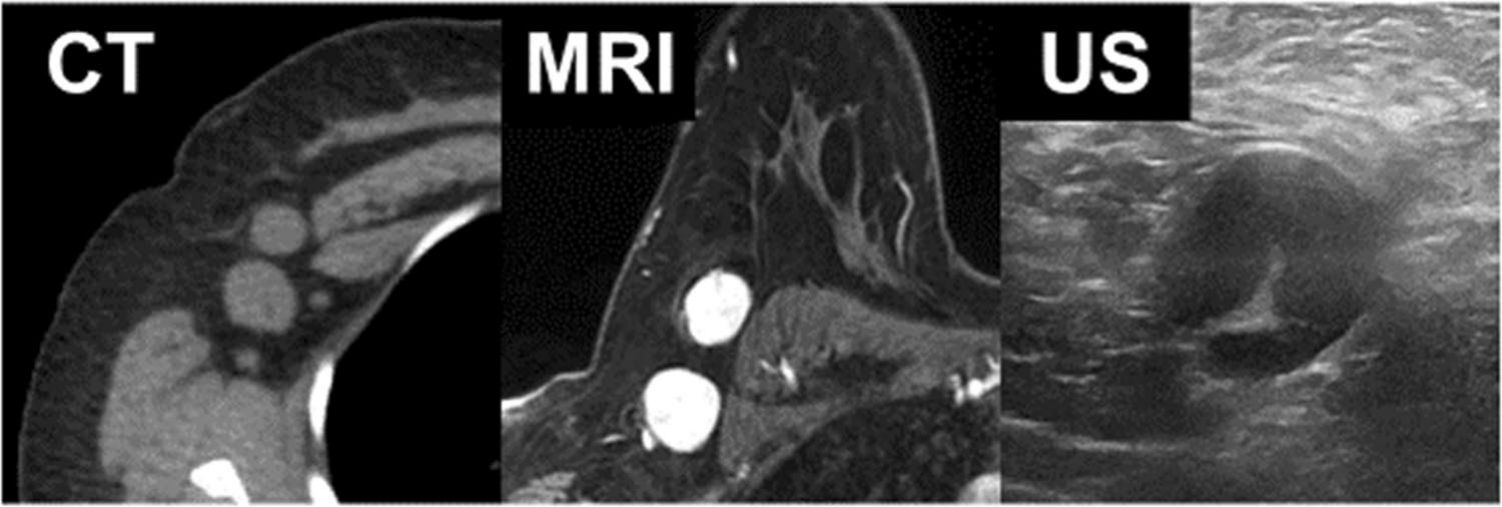
The same abnormal right axillary nodes by CT, MRI, and US in a breast cancer patient. All modalities demonstrate rounded rather than elongated shape, cortical thickening, and loss of the fatty hilum (a small residual fatty hilum is visualized by US)

CT, US, and MRI are often able to differentiate the lymph node cortex from the fatty hilum. Smaller microscopic features of lymph nodes, such as follicles and germinal centers, cannot be visualized by routine clinical imaging modalities. Thickening of the cortex or loss of the fatty hilum is an abnormal finding suspicious for malignancy (Fig. 12), although this may also indicate a node reactive to infection or inflammation.

**Fig. 13:**
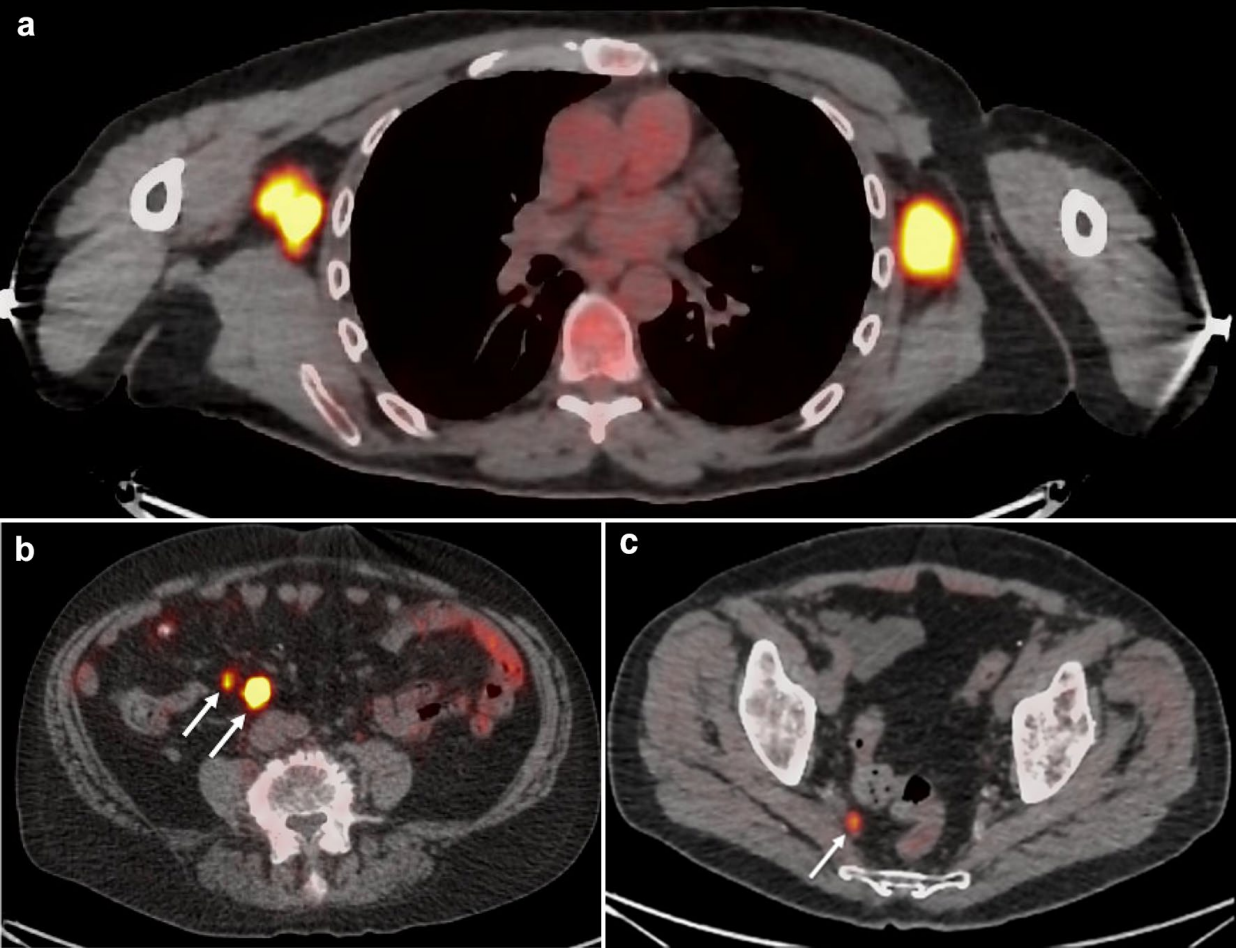
Axial fused PET-CT images of abnormal lymph nodes. FDG PET-CT (**a**) of bilateral axillary lymph node metastases from melanoma. DOTATATE PET-CT (**b**) of mesenteric nodal metastases from neuroendocrine tumor. Fluciclovine PET-CT (**c**) of a distal right internal iliac pelvic nodal metastasis from prostate cancer. Regardless of the radiotracer, the abnormal nodes appear as “hot spots.” For (b) and (c), the nodes are not large enough to be considered abnormal by CT alone

While clinical imaging modalities that are primarily anatomic can also provide functional information, such as color Doppler ultrasound of blood flow and diffusion-weighted MRI, PET and lymphoscintigraphy are purely functional modalities that are often correlated with anatomic imaging (usually CT). These are radiotracer techniques that detect and localize the accumulation of radiotracer within lymph nodes. The most common radiotracer for PET imaging is fluorine-18 fluorodeoxyglucose (FDG), a radiolabeled form of glucose that accumulates in tumor cells and other cells with increased glucose metabolism. Other commercially available PET radiotracers for tumor imaging include gallium-68 tetraazacyclododecanetetraacetic acid–DPhe1-Tyr3-octreotate (DOTATATE), a radiolabeled somatostatin analog for neuroendocrine tumors [39], and fluorine-18 anti-1-amino-3–18F-fluorocyclobutane-1-carboxylic acid (fluciclovine), a radiolabeled amino acid analog for prostate cancer [40]. Regardless of the radiotracer, positive lymph nodes on PET appear as “hot spots,” and correlation with CT or MRI provides localization and morphologic information (Fig. 13a–c). PET imaging of lymph nodes is most useful for identifying metastatic nodes before they appear enlarged or abnormal on anatomic imaging.

**Fig. 14:**
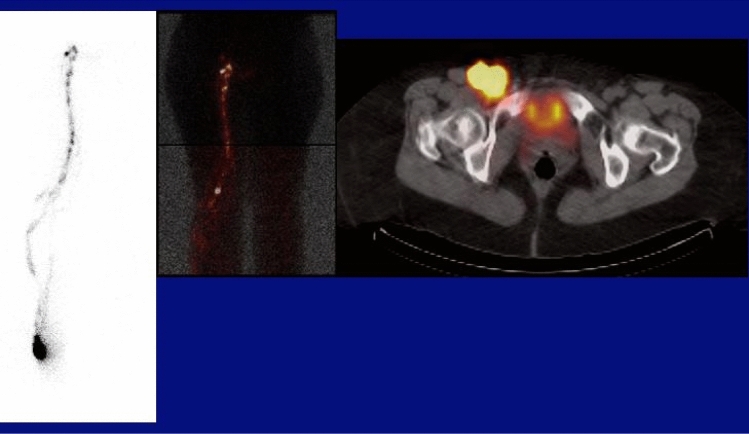
Lymphoscintigraphy with the injection site at the lateral right ankle. Activity is visualized in multiple lymphatic vessels in the right lower extremity on the partial whole-body acquisition (left). Delayed images fused with transmission images to show the body outline (middle) demonstrate a popliteal sentinel node in addition to multiple right inguinal nodes. SPECT provides cross-sectional images that can be registered to CT showing the “hot” right femoral SLN (right)

In lymphoscintigraphy, radiolabeled colloid or technetium-99 m tilmanocept are injected intradermally with uptake into lymphatic vessels and accumulation in lymph nodes for identification of SLNs. As in PET, the lymph nodes are visualized as “hot spots.” The lymphatic vessels may also be visualized as faint linear or curvilinear structures. Single photon emission computed tomography (SPECT) provides cross-sectional images that can be registered to CT (Fig. 14).

Anatomic imaging of lymph nodes by clinical modalities differentiates normal from abnormal based on size and morphologic characteristics. Functional imaging of lymph nodes demonstrates “hot spots” reflecting increased accumulation of radiolabeled glucose or other receptor-binding molecules.

## Application of sentinel lymph node surgery to different types of cancer

### Mark B Faries

Lymphatic mapping and SLN biopsy are now standard components of treatment in both melanoma [8] and breast cancer [9]. This is a natural development since both of these tumor types spread commonly along lymphatic pathways and lymph node metastases carry significant prognostic implications. If this is the case, it is reasonable to ask whether the same technique may be applicable in other cancer types. Indeed, SLN biopsy has been explored in other malignancies with varying levels of success and acceptance. In this section, the rationale for SLN biopsy as a general concept will be reviewed as well as the data related to its use in several non-melanoma, non-breast cancer malignancies (Table 1).

**Table 1:** Rationales for lymphatic mapping and sentinel lymph node biopsy

Rationale	Comment
Improve accuracy of staging	More intensive pathologic techniques (e.g. immunohistochemistry) can be applied since the number of nodes for evaluation is much smaller
Decrease morbidity of staging	Complete dissection of nodes is not required to obtain most staging information
Select patients for treatment of the entire basin	This might include complete surgical dissection, but may also involve radiation in diseases that are radiosensitive, such as Merkel cell carcinoma
Improve survival through early treatment of nodal metastases	This remains controversial and may depend on parts of tumor biology that are not well understood

First, what is the potential utility of the sentinel node concept? There are several possible ways in which SLN biopsy may be valuable including in increasing the accuracy of staging, in decreasing the extent of surgery and thereby its morbidity.

Lymphatic staging is a central component of prognostic assessment of many cancers, and involvement of reginal lymph nodes generally leads to a Stage 3 designation. However, in some cases the accuracy of pathologic staging may not be optimal with patients who were designated N0 suffering subsequent recurrence. One potential source of this is in microscopic metastases that are missed on standard pathologic evaluation. Application of more intensive pathologic techniques such as immunohistochemistry may improve the accuracy of pathologic assessment, but is generally impractical for large, complete nodal dissection specimens [41]. With SLN biopsy, however, the number of lymph nodes that require evaluation to provide an accurate pathologic assessment of the basin is much less, and the more exhaustive histopathologic techniques become feasible [42].

Beyond the assistance in pathologic assessment, SLN biopsy may also lead to more accurate identification of the relevant lymph nodes. Lymphatic drainage from a primary tumor site may be unpredictable in some cases. For cutaneous malignancies, drainage may occur to interval or ectopic nodal locations such as the epitrochlear or popliteal basins, the intermuscular triangular space, intracostal and retroperitoneal basins [43]. In other malignancies, such as colorectal cancer, drainage may occur to aberrant locations outside of the typical mesenteric location along the feeding vessels [44]. SLN biopsy has the ability to detect these unusual drainage patterns and locate the correct node(s) for pathologic assessment.

The second rationale for SLN biopsy is in order to decrease the extent and therefore the morbidity of surgical treatment. In melanoma and breast cancer, this is through avoidance of complete lymph node dissection [8, 45]. This particular benefit may be limited in some diseases, such as colorectal cancer where full dissections are done without significant additional morbidity. However, in other types of cancer, such as gastric cancer, it is possible that the accurate determination of the drainage pattern with tailoring of nodal dissection may reduce morbidity [46].

Survival improvement has been an additional goal of SLN biopsy and regional nodal surgery. This topic is much more controversial and a full discussion of the evidence for and against a survival impact will be left for another venue.

### Non-melanoma cutaneous malignancies

Other skin cancers harbor a real risk of nodal metastasis, including Merkel cell carcinoma and cutaneous squamous cell carcinoma. Merkel cell carcinoma is an uncommon diagnosis, but when it occurs lymph nodes are the most common site of initial metastasis [47], SLN biopsy has been employed successfully in staging regional lymph nodes. Merkel cell features including the diameter and depth of the primary tumor have been associated with the risk of nodal involvement, with larger tumors having a greater risk [48]. However, there has not been a subgroup that has been identified with less than a 10% risk of nodal involvement and current guidelines recommend consideration of SLN biopsy in all cases in patients who are candidates for surgery. The prognostic implications of a positive SLN in Merkel cell are not as significant as those in melanoma. This is due in part to the advanced age and comorbidities of many Merkel cell patients leading to increased risk of death from other causes. It does appear that SLN metastases are related to a higher risk of distant metastases [49]. The main utility of SLN biopsy in Merkel cell, though, is likely in selection of patients who should undergo radiation treatment to the reginal lymph nodes. It appears safe to spare radiation to that area for patients with negative SLNs in most cases. For those with SLN metastases, radiation without dissection appears to provide good regional disease control [50].

Cutaneous squamous carcinoma is an incredibly common diagnosis, and the vast majority of these cancers do not spread to lymph nodes. However, even a small minority of such common malignancy represent a large absolute number of cases, and there has been considerable interest in defining a group of patients who are at “high-risk” for nodal involvement and who should undergo SLN biopsy. Several staging systems are in current use including the 8^th^ Edition of the American Joint Commission on Cancer staging system and the Brigham and Women’s staging system. These systems do appear to identify patients who are at higher risk for recurrence and metastasis, but it is not clear that they can select patients for SLN biopsy. Additional research in this area will be useful to determine which patients would be most likely to benefit from the procedure.

Lymphatic metastases from colorectal cancer are also a common pathway of spread. Although full lymphatic dissection is a standard part of any colonic resection for cancer, lymphatic mapping may provide two advantages. First, aberrant drainage to a nodal location outside of the standard dissection field has been identified in up to 10% of cases [44]. Lymphatic mapping may identify these nodes that would normally be missed. Second, more intensive analysis of a small number of SLN may improve staging. This might identify additional stage III patients, but more importantly it may allow patients who are truly node negative to avoid chemotherapy [51]. However, outside of a few specialized centers, accurate mapping of colon cancer has been challenging and the technique remains investigational in most locations.

Nodal dissection in gastric cancer has been a source of considerable controversy worldwide. In Asia where there are particularly high rates of gastric cancer, considerable progress has been made in decreasing the morbidity and extent of surgery for patients with early stage disease. Lymphatic mapping has been incorporated into this progress, particularly in Japan [46, 52, 53] as mentioned earlier. The use of fluorescent tracers allows mapping in the context of laparoscopic and robotic procedures with lymph node dissections tailored to the drainage patterns of each individual primary tumor. This holds the promise of improving recovery and quality of life while maintaining accurate and complete staging and treatment.

Lung cancer is one of the most common and most deadly malignancies, and many patients suffer recurrence despite negative nodal staging at the time of surgery. More thorough evaluation of pulmonary lymph nodes has been associated with improved staging and outcomes [54]. Application of lymphatic mapping to lung cancer has been especially challenging due to the physically confined space of the thoracic cavity with nodes frequently in close proximity to the primary tumor. It is also more difficult to map with vital blue dyes due to the anthracotic nature of pulmonary lymph nodes. Recent series using fluorescent dyes and near infrared imaging have demonstrated outstanding survival among patients who are determined to be free of nodal metastases through SLN biopsy [55].

Lymph node staging is also important in gynecologic malignancies and nodal dissections are a standard component of treatment for many patients with ovarian, cervical and uterine cancers. However, lymphatic mapping and SLN biopsy have not become widely adopted. Research continues to define the role of SLN biopsy in those diseases. In ovarian cancer, the Sentinel-nodes in Early-Stage Ovarian Cancer Trial (SELLY) study used mapping with indocyanine green injection in the ovarian pedicle with a SLN detection rate of 68% and high sensitivity [56]. In cervical cancer, a recent meta-analysis including 21 studies and 2234 patients found lymphatic mapping with 88% side specific sensitivity. Side specific detection rates were similar with technetium-based or indocyanine green-based mapping [57]. In endometrial cancer, retrospective comparison of SLN biopsy to complete node dissection found fewer complications for SLN patients, particularly in relation to lymphedema [58]. Similar retrospective comparisons suggest equivalent or improved oncologic outcomes, though prospective clinical trial evaluation of this question is still lacking [59].

Finally, urologic cancer was one of the early areas of interest in the SLN concept [6]. In penile cancer, current guidelines allow SLN biopsy as an alternative to inguinal dissection in early stage disease, but in prostate cancer, the technique has not yet been widely accepted [60]. Technological innovation, particularly in tracers and imaging, hold promise to make the technique more accurate and useful in that disease [61].

Overall, it is clear that the procedure of mapping lymphatic drainage of a primary tumor site and evaluating the SLN(s) that are directly on those pathways provides a highly accurate picture of whether tumors have begun to spread via that route. The value of the procedure extends beyond melanoma and breast cancer where it has become a well-established standard. However, the value of applying the technique to other malignancies will depend on the biology of that tumor type, i.e. whether the tumor spreads primarily to regional lymph nodes as its first or primary mode, and the technical challenges of the anatomic locations of the tumors. These factors, together with the hard work of surgeons in clinical research, will determine how fast and how far SLN biopsy will extend its reach in oncology sampling in esophageal and gastric cancer.

## Tumor-associated lymphatic vessels and immune surveillance

### Amanda W. Lund

While much attention is paid to the contribution of the lymphatic vasculature to melanoma metastasis, it is important to remember that afferent lymphatic transport at steady state is critical for immune surveillance [62, 63]. Lymph transports antigens and dendritic cells (DC) from peripheral non-lymphoid tissues to lymph nodes where interactions with naïve lymphocytes activate adaptive immune responses. Initial evidence for the importance of lymphatic transport in immune surveillance in melanoma came from murine studies where the lymphangiogenic growth factor, vascular endothelial growth factor (VEGF-C), was overexpressed in melanoma cells and implanted into immune competent hosts [64]. While previous work demonstrated a clear link between VEGF-C overexpression, lymphangiogenesis, and lymph node metastasis [65, 66], the use of immune compromised animals in these experiments obviated the opportunity to investigate immunological implications. While VEGF-C overexpression in immune competent hosts remained pro-metastatic, it became further evident that tumor-associated lymphangiogenesis boosted fluid and DC transport to lymph nodes and intratumoral inflammation [64] leading to the hypothesis that lymphatic transport was not only necessary for immune surveillance but could be boosted for therapeutic benefit. Two additional studies investigating melanoma growth [63] and initiation [67] in mice lacking dermal lymphatic vessels supported this hypothesis, whereby the lack of a dermal lymphatic vasculature and associated afferent lymphatic transport significantly reduced tumor-associated inflammation, rates of tumor initiation and metastasis, and impaired the generation of de novo cytotoxic CD8^+^ T cell responses. The preclinical association between lymphatic vessels and tumor-associated inflammation has been explored to some extent in patient material [63, 68, 69], but more work remains to be done to understand the extent to which lymphatic vessel density is itself a surrogate for tumor-associated inflammation in patient samples.

**Fig. 15:**
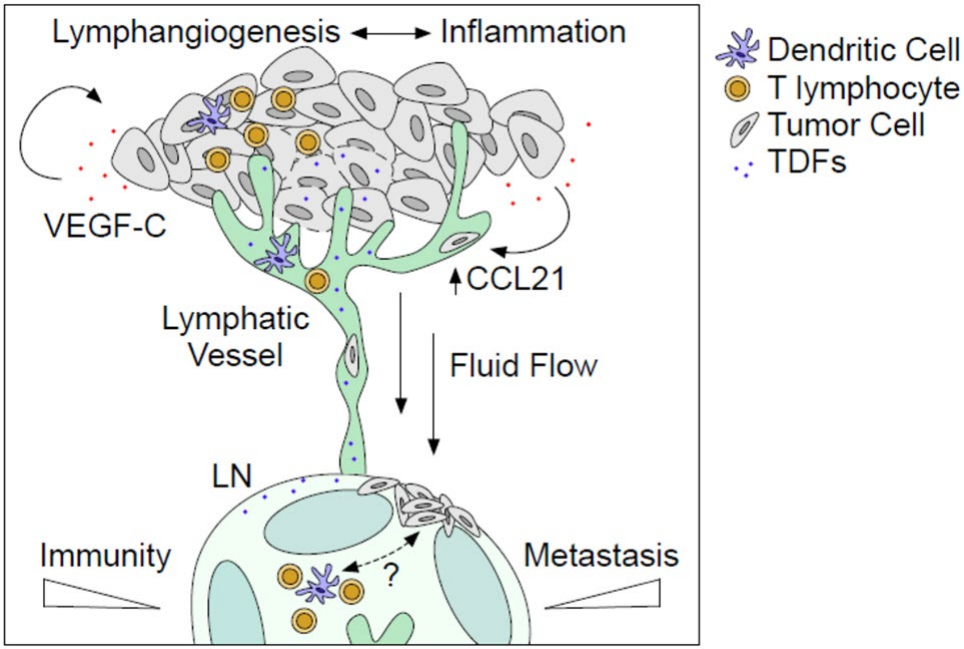
Overlapping mechanisms regulate melanoma immunity and metastasis. VEGF-C overexpression in tumor microenvironments induces tumor and lymph node (LN) lymphangiogenesis, which is associated both with the accumulation of intratumoral inflammation, antigen presentation, and LN metastasis. VEGF-C activates lymphatic proliferation and production of homing chemokines (e.g. CCL21) that recruit antigen presenting dendritic cells (DC), tumor cells, and T lymphocytes (memory, regulatory, and effector) out of tumors into afferent lymph and towards draining, sentinel LNs. In LNs, DCs interact with naïve T lymphocytes to initiate de novo, tumor-specific immune responses, but activation may be hindered by delivery of immune suppressive cytokines and tumor-derived factors (TDF) that drive LN immune suppression and support metastatic seeding and outgrowth. Furthermore, lymphatic endothelial cells harbor intrinsic immunomodulatory activity (e.g. antigen scavenging) that limits T cell expansion and function. The inefficiency of the immune response to cancer, therefore, likely depends on these overlapping mechanisms that both activate immune surveillance and mediate immune suppression and metastasis. Developing a specific, therapeutic paradigm to boost immune surveillance through lymphatic transport consequently must dissect the relative mechanisms of immunity and metastasis in vivo to identify therapeutic windows of opportunity for patient care

Based on this shift in paradigm, recent work took a therapeutic approach to ask whether VEGF-C overexpression could improve response to preclinical immunotherapy. In both murine melanoma [68] and glioblastoma [70] models, VEGF-C-induced inflammation primed tumors for responsiveness to immune checkpoint blockade and generated long lasting antigen-specific responses that established memory capable of reducing recurrence. The presumption from this work is that elevated lymphatic transport and DC migration to lymph nodes enhances presentation of tumor-associated antigens to the lymph node. Given the well documented role of VEGF-C in driving metastasis, however, and the overlapping mechanisms used by DCs and tumor cells to access afferent lymphatic vessels [71–73], the safety of a pro-lymphangiogenic therapeutic approach in patients remains to be determined (Fig. 15).

Importantly, the positive effects of immunotherapy in the setting of elevated lymphangiogenesis may be further tempered by immune suppressive events in tumor-draining lymph nodes [74], that likely results from factors delivered via lymph. To this point, VEGF-C overexpression in melanoma actually suppresses the de novo priming of CD8^+^ T cells specifically within tumor-draining lymph nodes, at least in part due to the unique scavenging and cross-presenting capability of lymphatic endothelial cells [64, 75]. Additionally, peripheral lymphatic capillaries and their lymph node counterparts express inhibitory immune checkpoints and may directly impair anti-tumor immune responses [76–78]. Consequently, the lymphatic vasculature harbors multiple overlapping mechanisms that likely converge in a tissue-specific manner to regulate anti-tumor immunity.

Finally, though much attention is directed at the DC as a critical mediator of immune surveillance, T lymphocytes are also abundant in lymph and the kinetics of their trafficking into and out of tumors remains largely unexplored. Through the use of in vivo photoconversion, recent studies indicate that CD8^+^, CD4^+^, and gamma delta T cells access tumor-associated lymphatic vessels to egress from tumor microenvironments [79, 80]. Recently egressed effector CD8^+^ T cells maintain their ability to produce cytotoxic cytokines in response to ex vivo stimulation [80] and may represent a therapeutically-relevant pool of cytotoxic T cells with tumor killing potential. While both the functional consequences of T cell egress and the mechanisms that regulate it remain unclear, continued work in this area may reveal new control points for the manipulation of in situ, effector immune responses for patient benefit. As the immunological implications of afferent lymphatic transport and lymphangiogenesis continue to emerge, future work will need to carefully evaluate the relative effects of lymphangiogenesis on immunity and metastasis to reveal safe and efficacious therapeutic strategies for future application.

## Summary and future perspectives

### Stanley P. Leong

For this review article, the anatomic basis of the lymphatic system for different sites is summarized by Alex Pissas et al. relating to the lymphatic system of the stomach and pancreas and by Miguel Amore for the upper and lower extremities. Max Wu describes the imaging of the lymphatic system and the lymph nodes. Further, Mark Faries addresses the concept of SLN first introduced in melanoma and then applied to other types of cancer. Beyond the anatomical description and physiological role of the lymphatic system including the SLNs, Amanda Lund discusses the molecular association between tumor and lymphatic vessels suggesting the important role of immune surveillance of tumor by the lymphatic system. While lymphatic vessels facilitate regional metastasis, they also play a critical role in immune surveillance. Better definition of the molecular mechanisms of lymphatic transport may point to new strategies to stop cancer metastasis while promoting anti-tumor immunity.

**Fig. 16:**
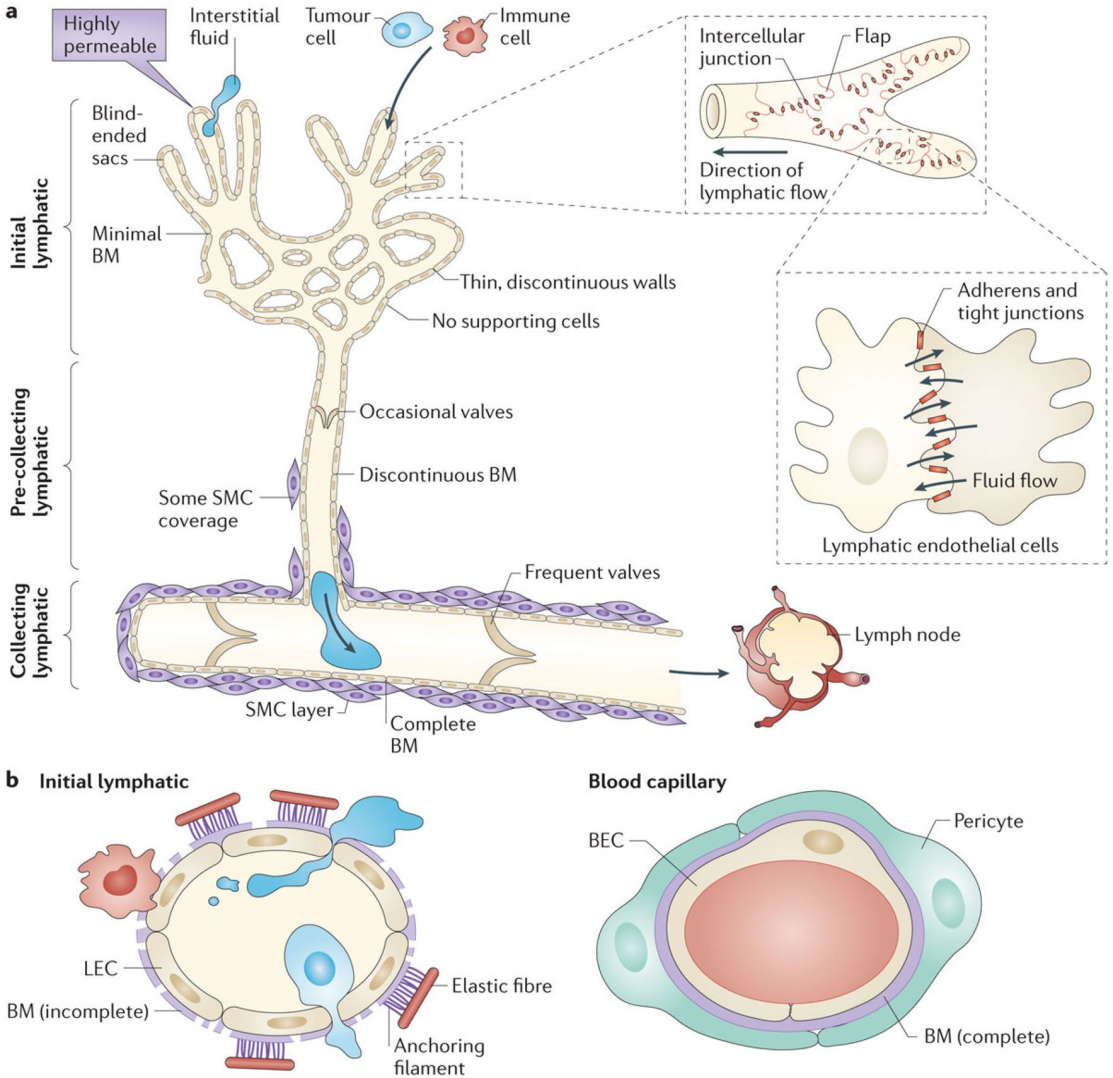
Structure of the lymphatic vasculature and of lymphatic endothelial cells from initial lymphatics. The hierarchy of lymphatic vessel subtypes is shown in part **a**, with the characteristics of the subtypes indicated. The association between lymphatic endothelial cells (LECs) in initial lymphatics is shown in the two pull-out boxes, with the contact regions between LECs in the vessels being indicated by the brown lines in the first pull-out box. Fluid is thought to enter (as shown by the arrows in the second pull-out box) via the tips of the flaps of LECs, without compromising the integrity of the intercellular junctions on the sides of the flaps. The structure of LECs from initial lymphatics is shown in part **b**, illustrating the entry of fluid and cells, which is favored when the interstitial fluid pressure is high, causing stretching of the extracellular matrix. The structure of a blood capillary is shown for comparison. The anchoring filaments that are associated with initial lymphatic vessels connect the abluminal membrane of LECs to surrounding elastic fibers—these filaments are short, but for visual clarity they are shown larger than to scale. Anchoring filaments are involved in controlling the entry of cells and interstitial fluid into the initial lymphatics. It should be noted that cells can cross lymphatic endothelial layers via a transcellular route, as well as via the paracellular route that is shown here. BEC, blood endothelial cell; BM, basement membrane; SMC, smooth muscle cell. Legends and figure are reprinted by permission from Springer Nature Customer Service Centre GmbH: Springer Nature [83]

The lymphatic system consists of the lymphatic vessels and lymph nodes and plays an important physiologic role in the body’s circulatory system as a major drainage system to return about 3 out of the 20 L of fluid (10–15%) from the interstitial space daily, containing waste products including cellular debris, macromolecules of proteins, excess water and toxins (Fig. 8) [81]. In addition, the lymphatic system also includes the mucosa-associated lymphoid tissues such as tonsils, adenoids, Peyer’s patches in the small bowel and even the appendix. The average diameter of the lymphatic vessel is 100 micron with uni-directional semi-lunar valves. The lymphatic vessels are thin-walled and the wall is lined by endothelial cells surrounded by smooth muscles with the outermost layer being the adventitia which anchor the lymphatic vessels to the surrounding tissue, primarily the adipose tissue [82]. Figure 16 shows the detailed drawing to illustrate the microanatomy of the structure of the lymphatic vessel [83]. Several molecular markers have been found and characterized in lymphatic endothelial cells but not blood endothelial cells. Thus, identification of lymphatic vessels has been made easy by immunofluorescence or immunohistochemical labeling. These molecular markers include the Drosophila melanogaster homeobox gene prospero, known as prospero homeobox protein 1 (PROX1 or Prox1; abbreviations for human and rodent proteins, respectively), hyaluronan receptor 1 of the lymphatic vessel endothelial cells (LYVE1 or Lyve1), vascular endothelial growth factor receptor-3 (VEGFR3), and podoplanin. These markers have proven useful to identify lymphatic vessels being used usually in combination with each other or with a pan-endothelial marker [82]. The lymphatic vessels drain into lymph nodes, a factory of immune cells such as T cells, B cells, dendritic cells and macrophages. There are about 600 lymph nodes in the body within the lymphatic system. The primary physiological role of the lymphatic system is to filter the body waste products and fight infection through innate and specific immunity. For a more detailed review of the anatomy and physiology of the lymphatic system, additional information is available in separate reviews [83–85].

Cancer cells exploit the lymphatic system as a conduit to travel from the primary site from their microenvironment and enter into the lymphatic vessels toward the lymph nodes and beyond to the distant sites. The average diameter of a lymphocyte is 15 microns, and the average diameter of a cancer cell is slightly larger than a lymphocyte, up to 20 microns or more. The size of a single cancer cell can comfortably travel within the 100 micron lymphatic vessel. It has been shown that lymphatic vessels induced by cancer by means of lymphangiogenesis may become dilated [86]. It is speculative that a cluster of cancer cells may choke in the lymphatic vessel at the valve of the lymphatic vessel [87] and eventually growth on the inner wall of the lymphatic vessel as a colony to grow into the surrounding fat tissue as in-transit metastasis, such as the case in melanoma. It may also be speculated that cancer cells may have a specific receptor to attach to the inner side of the lymphatic vessel and set up a residence.

This review article describes the anatomic basis of the human lymphatic system with emphasis of the potential routes of cancer spread through the lymphatic system. In a separate review article, molecular mechanisms of cancer metastasis via the lymphatic versus the blood vessels will be discussed in detail with emphasis in cancer lymphangiogenesis [88]. It is obvious from individual sections of this review article that the lymphatic system draining various body organs is not uniform, with the lymphatic channels draining the internal organs being more complex (Fig. 8) than the peripheral ones such as the skin and the breast. The SLN drainage for melanoma and breast cancer follow more reliable routes than the internal organs such as the lungs, stomach, pancreas and other organs, which show complicated lymphatic drainage patterns. Certainly, melanoma and breast cancer allow us to appreciate the biology of early cancer arriving in the SLNs with minimal tumor burden. It has been shown that melanoma patients with a negative SLN biopsy have low recurrence rate of 5–10% [89, 90]. For breast cancer patients with a negative SLN biopsy, their recurrence rate is also very low, less than 5% [91]. Overall, melanoma or breast cancer patients with a negative SLN biopsy fare much better than those with a positive SLN biopsy [92, 93]. Thus, it is important to learn the biology of cancer trafficking and lymph node metastasis in early melanoma and breast cancer to appreciate the mechanisms of cancer spread from the primary site to lymph nodes and beyond to the distant sites. Whether there are separate routes of spread from the primary site through the blood vessels other than the lymphatic system will be fully addressed in two review articles [88, 94]. The relative simplicity of melanoma and breast cancer metastasis through the SLNs, in contrast to the complicated lymphatic system of the stomach, pancreas and other organs, is akin to Mendel’s discovery of the independent segregation of the dominant and recessive genetic alleles in the pea model with respect to seven features of the peas including tall and short, smooth and wrinkled, green and yellow and others [95]. If Mendel would have used the human hair color variations to study the genetic model of heredity, he would not have arrived at these clear and discrete conclusions as there are multiple cross-over genes involving hair color heredity. Thus, the SLN model for melanoma and breast cancer, being relatively simple, will help us to understand the spread of cancer to the lymph node and its interaction within the lymphatic channels. The microenvironment within the primary tumor site and the SLN in relationship to the cancer growth and spread awaits further study [96].

## Data Availability

Data sharing not applicable to this article as no datasets were generated or analysed during the current review.

## Abbreviations

SLN: Sentinel lymph node
CT: Computed tomography
US: Ultrasound
MRI: Magnetic resonance imaging
PET: Positron emission tomography
DC: Dendritic cell
VEGF-C: Vascular endothelial growth factor
